# miRNA-148b suppresses hepatic cancer stem cell by targeting neuropilin-1

**DOI:** 10.1042/BSR20150084

**Published:** 2015-07-29

**Authors:** Qinying Liu, Yangmei Xu, Shenghong Wei, Wei Gao, Li Chen, Tong Zhou, Zhen Wang, Mingang Ying, Qiuhong Zheng

**Affiliations:** * Fujian Provincial Key Laboratory of Tumor Biotherapy, Teaching Hospital of Fujian Medical University, Fujian Provincial Cancer Hospital, Fuzhou 350014, P.R. China; † Department of Abdominal Surgery, Teaching Hospital of Fujian Medical University, Fujian Provincial Cancer Hospital, Fuzhou 350014, P.R. China; ‡ Department of Abdominal Tumor, Teaching Hospital of Fujian Medical University, Fujian Provincial Cancer Hospital, Fuzhou 350014, P.R. China; § Institute of Biomedical and Pharmaceutical Technology, College of Chemistry and Chemical Engineering, Fuzhou University, Fuzhou 350002, China

**Keywords:** cancer stem cell, hepatocellular carcinoma, microRNA-148b, Neuropilin-1, side population cell

## Abstract

The existence of cancer stem cells (CSCs) is considered as a direct reason for the failure of clinic treatment in hepatocellular carcinoma (HCC). Growing evidences have demonstrated that miRNAs play an important role in regulation of stem cell proliferation, differentiation and self-renewal and their aberrances cause the formation of CSCs and eventually result in carcinogenesis. We recently identified *miRNA-148b* as one of the miRNAs specifically down-regulated in side population (SP) cells of PLC/PRF/5 cell line. However, it remains elusive how *miRNA-148b* regulates CSC properties in HCC. In the present study, we observed that overexpression or knockdown of *miR-148b* through lentiviral transfection could affect the proportion of SP cells as well as CSC-related gene expression in HCC cell lines. In addition, *miR-148b* blocking could stimulate cell proliferation, enhance chemosensitivity, as well as increase cell metastasis and angiogenesis *in vitro*. More importantly, *miR-148b* could significantly suppress tumorigenicity *in vivo*. Further studies revealed that Neuropilin-1 (NRP1), a transmembrane co-receptor involved in tumour initiation, metastasis and angiogenesis, might be the direct target of *miRNA-148b*. Taking together, our findings define that *miR-148b* might play a critical role in maintenance of SP cells with CSC properties by targeting NRP1 in HCC. It is the potential to develop a new strategy specifically targeting hepatic CSCs (HCSCs) through restoration of *miR-148b* expression in future therapy.

## Abstract (precis)

Our study revealed that *miR-148b* was specifically down-regulated in hepatic cancer stem cells (HCSCs) and affected cell proliferation and metastasis in vitro and tumorigenicity in vivo by directly targeting to Neuropilin-1(NRP-1), a transmembrane co-receptor involved in metastasis, suggesting that enforced *miR-148b* expression might be an efficient therapeutic strategy to eradicate HCSCs and reduce metastasis.

## INTRODUCTION

Hepatocellular carcinoma (HCC) is known as the fifth most common cancer and the third cause of cancer-related mortality in the world. The existence of hepatic cancer stem cells (HCSCs) has been considered as the direct reason of clinical tumour relapse and therapeutic failure [1]. Increasing evidences have suggested that many miRNAs play critical roles in controlling the self-renewal and differentiation of stem cell and some of them were proved to express abnormally in HCSCs and regulate the key biological characteristics of HCSCs [2]. It suggests a new clue to conquer HCC by directly targeting and eradicating HCSCs through related-miRNA regulation [3]. However, it remains unclear the detailed mechanism about how miRNAs regulate the formation of HCSCs and maintain the properties of HCSCs.

Based on cancer stem cell (CSC) hypothesis, tumours consist of heterogeneous cell populations, in which only a small population called CSCs has the capability to drive tumour initiation [4]. Traditional surgery, chemotherapy and radiotherapy could eliminate the bulk of cancerous cells but hard to eradicate CSCs that maintain a high capacity for self-renewal, chemoresistance and pluripotency, leading HCC patients to suffer frequent recurrence or metastasis. Therefore, it is in urgent need of novel treatment strategies, which could target CSCs directly. Several functional assays have been developed to isolate CSCs, including side population (SP) approach, surface biomarker-based isolation, sphere formation and ALDEFLUOR-approach [1]. SP cells those can actively efflux Hoechst 33342 dye through ATP-binding cassette proteins (ABC) transporters have been proved to harbour CSC properties early in 2006 [5]. Compared with non-SP (NSP) cells, SP cells showed enhanced proliferation, chemoresistance and tumorigenicity in HuH-7 and PLC/PRF/5 cell lines, indicating that SP cells isolation is an effective method to identify HCSCs [5].

miRNAs are non-coding RNAs of ~19–23 nts that are crucial for many biological processes through regulating gene expression. Several miRNAs, which regulate differentiation of normal stem cells, are also involved in carcinogenesis. Ji et al. [6,7] revealed that the *miR-181* family are highly expressed in epithelial cell adhesion molecule (EpCAM)+AFP+ cells of HCC and maintained the CSC characteristics through targeting caudal type homeobox 2 (CDX2), GATA binding protein 6 (GATA6) and nemo-like kinase (NLK), which are essential for hepatic cell differentiation through regulating Wnt/β-catenin signal pathway. *miR-21* was also detected to overexpress in SP cells of HCC and increase the ability of migration and invasion via degradation of tumour suppressors phosphatase and tensin homolog (PTEN), reversion-inducing-cysteine-rich protein with kazal motifs (RECK) and programmed cell death 4 (PDCD4) [8]. Besides these oncogenic miRNAs, more miRNAs were found to be specifically down-regulated in CSC of HCC as tumour suppressors, such as *miR-142-3p, miR-150, miR-145, miR-612, miR-200a* and *miR-200c*. They contribute to cell proliferation, drug resistance, cell migration and invasion, angiogenesis and tumorigenesis both *in vitro* and *in vivo* [9–14]. To target and suppress HCSCs by regulating these miRNAs may shed light on the development of efficient therapeutic strategies against HCC. However, it remains a substantial need for more thorough investigation on the role of miRNAs in maintenance of CSC-like properties and regulation of tumour initiation and progression.

In previous study, we performed the quantitative real-time (qRT)-PCR profile and detected 27 miRNAs aberrantly expressed in SP cells of PLC/PRF/5 cell line, including *miR-148b*, miR-9*, *miR-194* and so on [15]. We noticed that *miR-148b* was one of the most down-regulated miRNAs, suggesting that it might function as a key regulator in SP cells. Coincidentally, Liu et al. [16] also detected differently miRNAs expression profile between SP and NSP F344 rat HCC cell and found out 10 under-expressed miRNAs in SP cells, which contained miR-148b*. Moreover, accumulating evidences support that *miR-148b* acts as a tumour-suppressor in various tumour types, such as breast cancer, lung cancer, pancreatic cancer, gastric cancer, colorectal cancer and non-Hodgkin's lymphoma [17–22]. A clinical research recently revealed that *miR-148b* was significantly decreased in HCC and involved in tumour invasion and progression, based on the qRT-PCR data from 156 cases of HCC and 36 cases of normal control specimens [23]. Zhang et al. [24] also confirmed that *miR-148b* was significantly down-regulated in human HCC tissues and correlated with larger tumour size, more tumour number, metastasis and worse prognosis. Overexpression of *miR-148b* inhibited cell proliferation, tumorigenicity and cell invasion in HepG2 by targeting the gene wingless-type MMTV integration site family, member 1 (WNT1) [24]. Nevertheless, how *miR-148b* regulates the formation and maintenance of SP cells with CSC properties in HCC is still poorly characterized.

In the present study, we proved that *miR-148b* was down-regulated in SP of PLC/PRF/5 and HuH-7 cell lines. Gain-of-function and loss-of-function studies via lentivirus transfection could change SP population and relative CSC properties, including cell proliferation, drug-resistance, cell migration and invasion, angiogenesis and tumorigenicity. Dual luciferase assay revealed that Neuropilin-1 (NRP1), a membrane-bound co-receptor to tyrosine kinase receptors for vascular endothelial growth factor (VEGF), transforming growth factor-β1 (TGF-β1), hepatocyte growth factor (HGF), the platelet-derived growth factor homodimer (PDGF-BB) and Semaphorin-3 (SEMA3) family members [25], was the direct target of *miR-148b*. In conclusion, we suggested that *miR-148b* might play a critical role in maintaining SP cells with CSC properties by regulating NRP1 in HCC and enforced *miR-148b* expression might be an efficient therapeutic strategy to eradicate HCSCs.

## MATERIALS AND METHODS

### Cell culture

Human liver cancer cell lines PLC/PRF/5 and HuH-7 were purchased from the Cell Resource Center, Shanghai Institute of Biochemistry and Cell Biology at the Chinese Academy of Sciences. Cells were cultured in Dulbecco's modified Eagle's medium (DMEM; Gibco) containing 10% FBS and incubated at 37°C in a humidified environment containing 5% CO_2_.

### SP cell detection and isolation by flow cytometry

The cells were harvested, washed by PBS and suspended at 1×10^6^ cells/ml in Hank's balanced salt solution supplemented with 3% FBS and 10 mM Hepes. Cells were incubated at 37°C for 90 min with 15 μg/ml Hoechst 33342 (Sigma) alone or in the presence of 50 μM verapamil (Sigma). During the incubation, the tubes were shaken up and down every 20 min to mix the cells with the solution. The cells were washed by Hank's solution for twice, added with 1 μg/ml propidium iodide (Sigma) and then filtered through a 70-μm cell strainer (BD). SP cell analysis and sorting were performed using MoFlo XDP (Beckman Coulter). Hoechst 33342 was excited with the UV laser at 350 nm and fluorescence emission was measured with 450 (Hoechst blue) and 570 (Hoechst red) optical filters. Propidium iodide labelling was measured for the discrimination of dead cells.

### miRNA interference

Lentiviral *miR-148b*-Ubi-EGFP-multiple cloning sites (MCS) (*miR-148b*) and its control Ubi-EGFP-MCS (miR-NC), *miR-148b*-H1-MCS-cytomegalovirus (CMV)-EGFP (Inh-148b) and its control H1-MCS-CMV-EGFP (Inh-NC) were constructed by Genechem Company and transfected to the PLC/PRF/5 and HuH-7 cells according to the manufacturer's instructions, in the presence of virus at a multiplicity of infection of 10.

### RNA extraction and quantitative real-time PCR

Total RNA from cultured cells was extracted using TRIzol reagent (Invitrogen). qRT-PCR assays were carried out to detect mRNA expression using the GoScript Reverse Transcription System (Promega) and QuantiFast SYBR Green PCR Kit (Qiagen) according to the manufacturer's instructions. miRNA from cultured cells was extracted using miRNeasy Mini Kit (Qiagen). qRT-PCR assays for miRNA detection were carried out using miScript II RT Kit (Qiagen) and miScript SYBR Green PCR Kit (Qiagen) according to the manufacturer's instructions. The primers for *miR-148b* and the internal control U6 were purchased from Qiagen. The primers for gene cluster of differentiation 133 (CD133), EpCAM, ATP-binding cassette, sub-family G (WHITE), member 2 (ABCG2), matrix metallopeptidase 9 (MMP9), tissue inhibitor of metalloproteinase-1 (TIMP-1), BMI1 proto-oncogene (BMI 1), snail family zinc finger 1 (SNAI 1), twist-related protein 1 (TWIST 1), cadherin 1 (CDH 1), CDH 2, NRP1 and control β-actin are shown in Table 1.

**Table 1:** Primer for the genes used in the present study

Gene	Forward primer	Reverse primer
CD133	5′-CCTATAGAACAATCACTGAGC-3′	5′-CAGAGGAAGTATTGTTTGTG-3′
EpCAM	5′-GCTGGTGTTATTGCTGTTA-3′	5′-CTTCTCATACTTTGCCATTC-3′
ABCG2	5′-CTGTGAGGCCTATAATAACC-3′	5′-GAGTGGCTTATCCTGCTT-3′
MMP9	5′-CCTGGAGACCTGAGAAC-3′	5′-CAGGGACAGTTGCTTCT-3′
TIMP-1	5′-ACTCTTGCACATCACTACCT-3′	5′-AAACACTGTGCATTCCTC-3′
BMI 1	5′-CATTTTCTGCTGAACGACT-3′	5′-AGTGATCTTGATTCTCGTTG-3′
SNAI 1	5′-AGGTGTGACTAACTATGCAA-3′	5′-CATTACTCACAGTCCCTTTT-3′
TWIST 1	5′-ACCTAGATGTCATTGTTTCC-3′	5′-CTGTCTCGCTTTCTCTTTTA-3′
CDH 1	5′-CTGGTTAGTGATGCAGTTAG-3′	5′-GAACCTTCTGATGCTAAAGT-3′
CDH 2	5′- GGTTTGCCAGTGTGACT-3′	5′-GATAAGCAGGATGATGATG-3′
NRP1	5′-ACAGCTTCTTCCCAGTATAG-3′	5′-GTATCCACTCTCGGTAGGA-3′
β-Actin	5′-GTTACACCCTTTCTTGACAA-3′	5′-CTTCACCGTTCCAGTTTT-3′

### Soft agar colony formation assay

Make 0.6% agar–10% FBS DMEM bottom layer and 0.3% agar–10% FBS DMEM top layer with 5000 cells in each well of six-well plate. After 14 days incubation, colonies were fixed with methanol and stained with 0.1% Crystal Violet solution for 15 min and photographed.

### Cell migration and invasion assay

Cell migration and invasion experiment was assessed using the Millipore 24-well Millicell Chamber of pore size 8 mm (Millipore). For the migration assay, a total of 1×10^5^ cells in DMEM without FBS were added into the upper chamber of the insert. For the invasion assay, 2×10^5^ cells in DMEM without FBS were added into the upper chamber pre-coated with Matrigel (Sigma). DMEM with 30% FBS was in the lower chamber. After incubated for 24 h, chamber was fixed and stained with 0.5% crystal violet for 30 min and the non-invading cells were removed with cotton swabs. The number of invasive cells on the lower surface of the chamber membrane was then counted under a microscope at a magnification of ×400 in five random fields.

### Luciferase assay

PLC/PRF/5 cells were seeded in 96-well plates. After 24 h, 100 ng of pmirGLO-NRP1 3′-UTR [wild-type (WT)] or pmirGLO-mutant NRP1 3′-UTR [mutant-type (MUT)] were co-transfected into cells with *miR-148b* or miR-NC by Lipofectamine 2000. Forty-eight hours later, Firefly and Renilla luciferase activities were measured with a Dual-Luciferase Reporter System (Promega). The Renilla luciferase activities were used as an internal control for transfection efficiency. All experiments were repeated for three times.

### Western blot

Cells were harvested, washed and lysed by lysis buffer. Proteins were separated by SDS/PAGE (8% gel) and transferred to nitrocellulose membrane. After blocking in 5% non-fat milk for 1 h, the membranes were incubated with the following primary antibodies:rabbit anti-NRP1 monoclonal antibody (1:1000, Cell Signaling Technology), mouse anti-α tubulin monoclonal antibody (1:1000, Abcam). The proteins were visualized with enhanced chemiluminescence reagents (Pierce).

### Xenograft tumour model

Every group contains 10 male BALB/c nude mice of 6-week-old. Group 1 were injected with 5×10^6^ control miR-NC or *miR-148b* PLC/PRF/5 cells in 100 μl of PBS into either shoulder, whereas group 2 were injected with 5×10^6^ control Inh-NC or Inh-148b PLC/PRF/5 cells in 100 μl of PBS into either shoulder. Tumour volumes were measured with slide caliper every three days until scarification after 6 weeks. The tumours formed in BALB/c nude mice were fixed in formalin and embedded in paraffin. The sections were subjected to hematoxylin and eosin (H&E) staining or stained with anti-NRP1 antibody.

### Statistical analysis

All results are expressed as mean±S.D. Comparisons between groups were conducted with the unpaired *t* test. The relationship between *miR-148b* and NRP1 (CST) expression was explored by Spearman correlation. Values were considered to be significantly different at *P*<0.05. All statistical analysis was conducted using SPSS 13.0 software.

## RESULTS

### SP cells of HCC cell lines harbour cancer stem cell-like properties

SP cell assay based on actively efflux of Hoechst 33342 dye was reported to be an effective method to identify CSCs in HCC [5]. To investigate the characteristics of SP cells in HCC, we first detected and isolated SP and NSP cells from PLC/PRF/5 and HuH-7 cell lines by flow cytometry. As the data showed, the percentage of SP was 0.74±0.08% in PLC/PRF/5 and 0.77±0.09% in HuH-7 cells in the presence of Hoechst 33342, whereas the SP cells were diminished in both cell lines in the presence of the calcium channel blocker verapamil and Hoechst 33342 (Figure 1A). We further tested the expression of HCC-related CSC markers, including CD90, CD133, CD44, EpCAM and ABCG2, on the surface of SP and NSP cells by flow cytometry. The percentage of PLC/PRF/5 cell positive for CD90, CD133, CD44, EpCAM and ABCG2 was 2.21%, 44.11%, 4.63%, 53.02% and 67.12% in SP and 0.43%, 21.03%, 1.52%, 21.66% and 20.48% in NSP respectively (Figures 1B and 1C). Similarly, the percentage of HuH-7 cell positive for CD133, CD44, CD90, EpCAM and ABCG2 was 1.42%, 44.42%, 2.44%, 89.20% and 3.11% in SP and 0.88%, 23.77%, 1.40%, 81.81% and 1.83% in NSP respectively (Figures 1B and 1C). The results indicated that all of the five CSC markers were expressed much higher on the surface of SP compared with NSP cells in HCC cell lines. Additionally, sphere formation assay data showed SP cells could form much larger and more spheres than NSP cells after one-week culture in serum-free medium (Figure 1D), confirming that our sorted SP cells harbour CSC-like properties.

**Figure 1:**
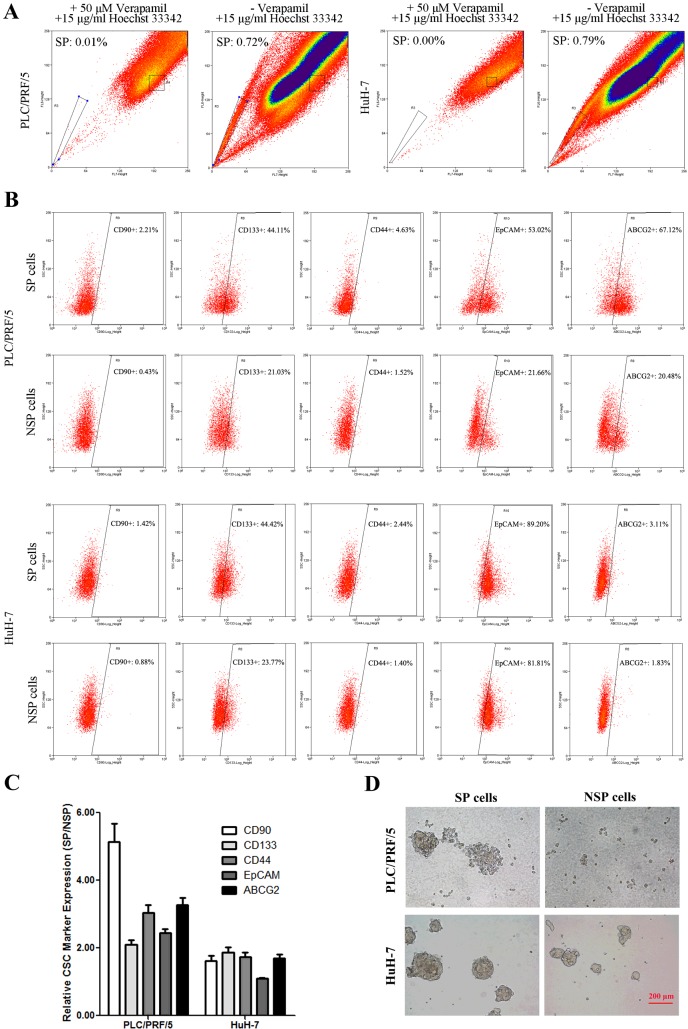
SP cell isolation and CSC-related properties analysis (**A**) PLC/PRF/5 and HuH-7 cells were incubated with 15 μg/ml Hoechst 33342 alone or in the presence of 50 μM verapamil and analysed by flow cytometry. (**B**) SP and NSP cells were immunostained with CSC-related marker CD90, CD133, CD44, EpCAM and ABCG2 and analysed by flow cytometry. (**C**) The relative CSC-related marker expressed on the surface of SP and NSP cells were showed by the histogram. (**D**) SP and NSP cells of PLC/PRF/5 and HuH-7 were cultured in serum-free medium for 2 weeks and observed under microscopes. Bar=200 μm.

### *miR-148b* was significantly down-regulated in SP cells in HCC cell lines

In previous study, we identified *miR-148b* as one of the 27 differentially expressed miRNAs between SP and NSP cells from PLC/PRF/5 [15]. In the present study, to re-verify the result in both PLC/PRF/5 and HuH-7 cell lines, we sorted SP and NSP cells separately by MoFlo XDP and analysed their *miR-148b* expression by qRT-PCR. As the results showed, the fold change of SP/NSP was −3.16±0.26 in PLC/PRF/5 and −3.58±0.30 in HuH-7 cells respectively (Figure 2A), similar with our previous study (SP/NSP=−3.14).

**Figure 2:**
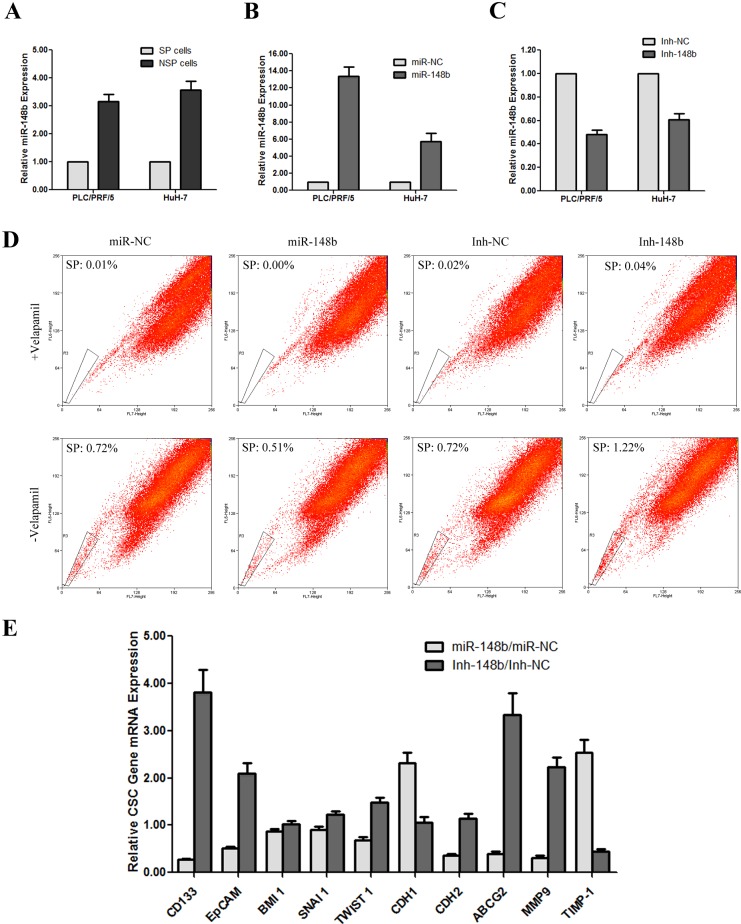
*miR-148b* was down-regulated in SP cells and affected the proportion of SP and the CSC-related gene expression in HCC cell lines (**A**) *miR-148b* was down-regulated in SP cells of both PLC/PRF/5 and HuH-7 cell lines. (**B**) qRT-PCR analysis of *miR-148b* overexpression cells (*miR-148b*) and its control cells (miR-NC) transfected by lentivirus in both PLC/PRF/5 and HuH-7 cell lines. (**C**) qRT-PCR analysis of *miR-148b* knockdown cells (Inh-148b) and its control cells (Inh-NC) transfected by lentivirus in both PLC/PRF/5 and HuH-7 cell lines. (**D**) *miR-148b* affected the proportion of SP cells in PLC/PRF/5. E. *miR-148b* affected the CSC-related gene expression in PLC/PRF/5.

### *miR-148b* expression affects the percentage of SP cells and the CSC-related gene expression in PLC/PRF/5

In order to further investigate the role of *miR-148b*, we established *miR-148b* stable overexpressing and inhibiting cell lines with lentivirus transfection. As shown is Figure 2(B), *miR-148b*-Ubi-EGFP-MCS (*miR-148b*) transfection increased the expression of *miR-148b* by 13.36- and 5.7-fold in PLC/PRF/5 and HuH-7 respectively. Conversely, *miR-148b*-H1-MCS-CMV-EGFP transfection (Inh-148b) reduced the expression of *miR-148b* by 2.08- and 1.64-fold in PLC/PRF/5 and HuH-7 respectively (Figure 2C).

Through SP cells detection by flow cytometry, we found that the fraction of SP in PLC/PRF/5 was increased from 0.70±0.08% to 1.24±0.11% when *miR-148b* was knocked-down, whereas the percentage of SP cells was decreased from 0.72±0.06% to 0.50±0.03% when *miR-148b* was overexpressed (Figure 2D), indicating that the expression level of *miR-148b* was tightly related with SP population maintenance in HCC.

We further examined whether *miR-148b* could affect the expression of CSC-related genes by qRT-PCR. As shown in Figure 2(E), *miR-148b* inhibition lead to a significant expression change of CD133 (increased 3.81 times), EpCAM (increased 2.10 times), ABCG2 (increased 3.33 times), MMP9 (increased 2.22 times) and TIMP-1 (decreased 2.28 times). Unexpected, the level of BMI 1, SNAI 1, TWIST 1, CDH1 and CDH2 kept almost unchanged, compared with the control Inh-NC cells. On the other hand, *miR-148b* overexpression did not affect the expression of BMI 1, SNAI 1 and TWIST 1 at all, but showed expression change of the other genes, including CD133 (decreased 3.74 times), EpCAM (decreased 1.95 times) and ABCG2 (decreased 2.53 times), MMP9 (decreased 3.25 times), TIMP-1 (increased 2.54 times), CDH1 (increased 2.32 times) and CDH2 (decreased 2.78 times), relative to control miR-NC cells. These findings suggested that the expression of *miR-148b* might inhibit the CSC properties and metastasis ability of cells in HCC.

### *miR-148b* inhibits cell proliferation and enhances chemosensitivity in PLC/PRF/5

Using real-time cellular analysis (RTCA), we found that the doubling time of Inh-148b cells from 80 to 120 h (46.93±1.01 h) was significantly shortened compared with that of control Inh-NC cells (67.80±1.08 h), whereas the doubling time of Inh-148b cells from 0 to 40 h and 40 to 80 h was not different from that of control Inh-NC cells in PLC/PRF/5 (18.39±4.45 compared with 16.04±3.28 and 24.85±0.13 compared with 24.43±0.03 respectively; Figure 3A). The data suggested that the knockdown of *miR-148b* could stimulate cells to keep growth even when culture environment changes to poor and unavailable. However, *miR-148b* overexpression seemed not to affect the cell proliferation as the doubling time of *miR-148b* and control miR-NC cells showed almost no difference during the whole culture time.

**Figure 3:**
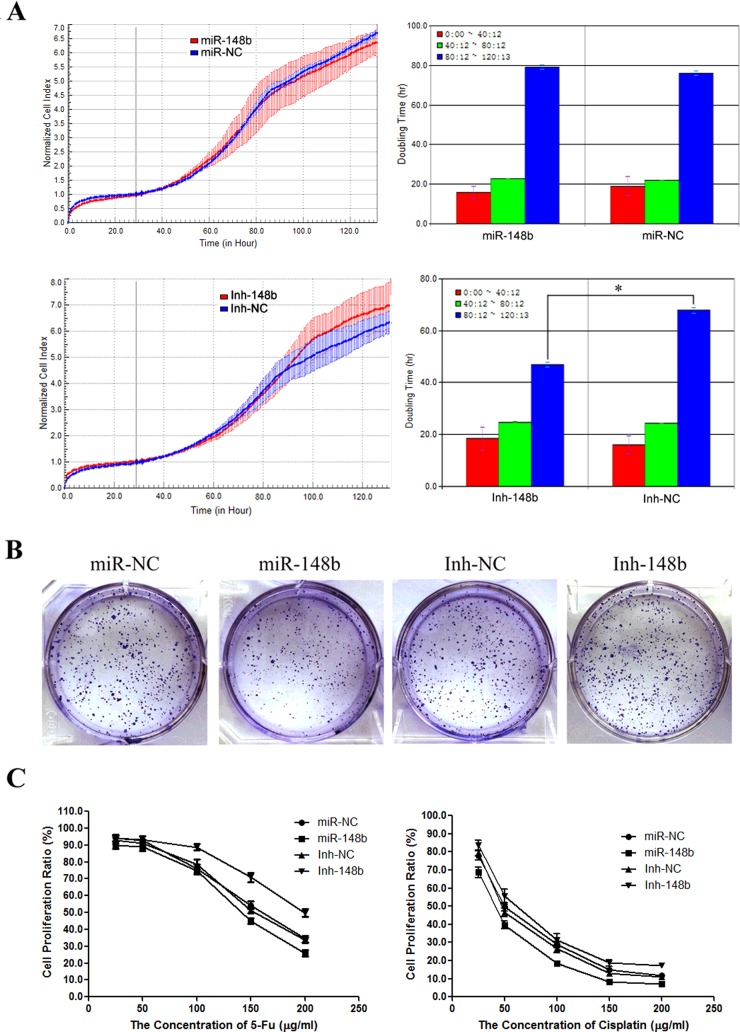
*miR-148b* inhibition stimulated cell proliferation and enhanced chemosensitivity (**A**) The detection of cell doubling time of *miR-148b*, miR-NC, Inh-148b and Inh-NC PLC/PRF/5 cells by RTCA. (**B**) Soft agar colony formation by *miR-148b*, miR-NC, Inh-148b and Inh-NC PLC/PRF/5 cells. (**C**) Chemosensitivity of *miR-148b*, miR-NC, Inh-148b and Inh-NC PLC/PRF/5 cells to 5-FU and cisplatin.

We further performed soft agar colony formation assay and observed that Inh-148b cells displayed more colonies compared with control Inh-NC cells; conversely, *miR-148b* cells displayed fewer colonies than miR-NC cells (Figure 3B). The finding indicated that the *miR-148b* blocking significantly stimulated cell proliferation.

To examine the effect of *miR-148b* on chemosensitivity of cells in HCC, we treated *miR-148b*, miR-NC, Inh-148b and Inh-NC PLC/PRF/5 cells with gradient concentrations of 5-Fu or cisplatin, which were two of the most commonly used traditional chemotherapy agents in clinic for HCC and tested the alternation of cell proliferation by WST-1 assay. As expected, the IC50 of Inh-148b was significantly increased under the treatment of 5-Fu (202.7 μg/ml compared with 154.8 μg/ml) or cisplatin (61.49 μg/ml compared with 50.60 μg/ml), whereas the IC50 of *miR-148b* was decreased under the treatment of 5-Fu (139.7 μg/ml compared with. 157.7 μg/ml) or cisplatin (39.57 μg/ml compared with 52.69 μg/ml; Figure 3C).

### *miR-148b* inhibits migration, invasion and angiogenesis of PLC/PRF/5 cells

We further performed scratch wound healing assay and transwell assay to test the cell migration and invasion ability in *miR-148b* gain-of-function or loss-of-function cells. The scratch wound healing assay results showed that 24 h after the wound was created, the gap area of Inh-148b cells reduced to 58.1±2.9% whereas that of control Inh-NC cells only reduced to 76.8±2.3%; conversely, the gap area of *miR-148b* cells after 24 h almost had no change (Figure 4A), confirming that *miR-148b* expression abolished the cell migration ability. The transwell assay results also showed that compared with Inh-NC cells, Inh-148b raised the cell invasion ability by 1.84±0.11-fold, whereas *miR-148b* decreased the ability of invasion by 1.50±0.07-fold (Figure 4B), indicating that *miR-148b* could suppress metastasis in HCC. Moreover, the effect of *miR-148b* on the formation of capillary tubes in matrigel was also assessed. As the data showed *miR-148b* PLC/PRF/5 cells reduced the capillary tube length by 1.53±0.08-fold and area by 1.78±0.07-fold; in contrast, blocking *miR-148b* increased both the tube length by 1.16±0.04-fold and the tube area by 1.77±0.06-fold (Figure 4C), suggesting that *miR-148b* could inhibit the capillary-like structure formation. Collectively, our results indicated that *miR-148b* could suppress migration, invasion and angiogenesis in PLC/PRF/5.

**Figure 4:**
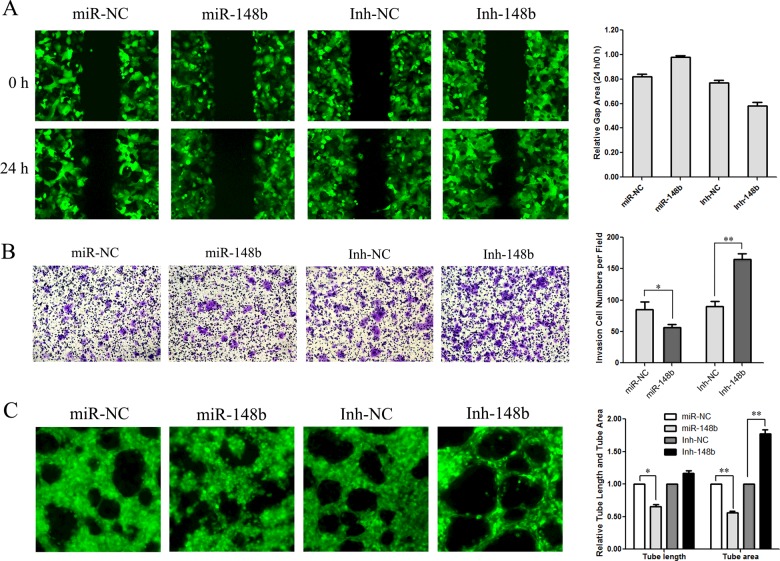
*miR-148b* affected cell migration, invasion and angiogenesis *in vitro* (**A**) *miR-148b* affected scratch wound healing. (**B**) *miR-148b* affected cell invasion by transwell assay. (**C**) *miR-148b* affected the tube formation in matrigel.

### *miR-148b* suppresses tumorigenicity of PLC/PRF/5 *in vivo*

To further confirm the function of *miR-148b* in tumorigenicity, an *in vivo* tumour xenograft model was used. Ten nude mice with 6-week-old were injected with *miR-148b* (right side) and control miR-NC (left side) PLC/PRF/5 cells in both shoulders (Group 1) and other ten nude mice were injected with Inh-148b and control Inh-NC PLC/PRF/5 cells in both shoulders too (Group 2). After 6 weeks, nine mice were left in Group 1, whereas only four mice were left in Group 2; the rest died because of non-controllable factors. As shown in Figures 5(A)–5(D), the tumours formed by *miR-148b* cells injection were much smaller than those formed by miR-NC cells injection (20.1±18.7 mg compared with 226.7±155.6 mg (*P*<0.05), as well as the tumour growth rate of *miR-148b* cells was much slower than that of miR-NC after injection. On the other hand, the mean tumour weight of mice injected with Inh-148b cells was remarkably higher than that injected with control Inh-NC cells (518.4±310.7 mg compared with 56.1±76.9 mg; Figures 5E–5H) and the tumour growth rate of Inh-148b was much faster than that of Inh-NC after injection. The results strongly suggested that *miR-148b* could inhibit the tumorigenicity of PLC/PRF/5 *in vivo*. Moreover, through analysing the SP percentage of tumour formed by injection of *miR-148b* or Inh-148b PLC/PRF/5 cells, we found that the tumour formed by Inh-148b cells contained 5.29% SP cells, whereas the compared tumour formed by Inh-NC cells only contained 2.44% SP cells; conversely, *miR-148b* tumour only contained 1.59% SP cells, which was much less than miR-NC tumour contained SP cells (3.10%; Figure 5I), indicating that *miR-148b* could stimulate the SP cell formation *in vivo*.

**Figure 5:**
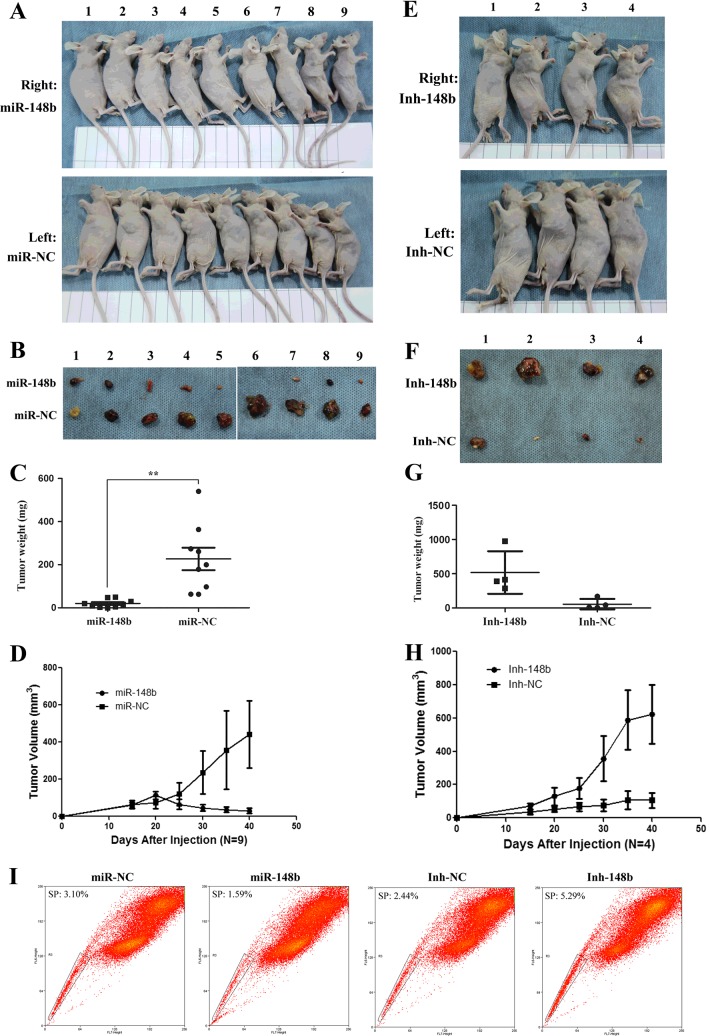
*miR-148b* suppressed the tumorigenicity *in vivo* (**A**) Six-week-old male nude mice were injected by 5×10^6^ control miR-NC or *miR-148b* PLC/PRF/5 cells in either shoulder (Group 1). Six weeks later, the mice were killed. (**B**) The tumours were showed. (**C**) The weight of tumours was analysed by statistics. (**D**) The growth of tumour volume after injection. (**E**) Six-week-old male nude mice were injected by 5×10^6^ control Inh-NC or Inh-148b PLC/PRF/5 cells in either shoulder (Group 2). Six weeks later, the mice were killed. (**F**) The tumours were showed. (**G**) The weight of tumours was analysed by statistics. (**H**) The growth of tumour volume after injection. (**I**) The SP fraction percentage of tumour formed in mice was analysed by flow cytometry. The weight and volume of miR-NC and Inh-NC tumours were different partly because the sample sizes for the two nude mice groups were different (10 nude mice for Group 1; only four nude mice for Group 2 and the other six mice died because of too heavy tumour burden) and partly because the growth of the miR-NC or Inh-NC tumour was affected by the tumour of *miR-148b* or Inh-148b on the other side shoulder of the same mouse. Especially the huge tumours of Inh-148b competed and obtained much more nutrition and resources and lead the tumours of Inh-NC on the other side shoulders to be smaller than expectation.

### *miR-148b* targets to NRP1 directly

To explore the function of *miR-148b*, we used three computational algorithms, including TargetScan, miRNA and miRDB (a microRNA target predietion and functional annotation database), to identify the target genes of *miR-148b*. Among the predicted targets, we focus on gene NRP1 since it was reported to be involved in induction of epithelial-to-mesenchymal transition (EMT) and maintenance of CSC phenotype [25]. According to the prediction by the seed complementarity, a potential *miR-148b*-binding site was found at nt 2135–2142 of NRP1 3′-UTR. The sequence alignment of *miR-148b* and the 3′-UTR of NRP1 transcripts was shown in Figure 6(A). To verify the direct interaction between *miR-148b* and NRP1, we cloned 3′-UTR sequences of NRP1 that contain predicted target site (WT) and seed-region-mutated sequences (MUT) of *miR-148b* into the pmirGLO vector respectively (Figure 6B). The results showed that co-transfection of *miR-148b* lentivirus significantly decreased the firefly luciferase activity of the reporter with WT 3′-UTR of NRP1, but not that of the mutant reporter, which indicates that *miR-148b* could directly target the 3′-UTR of NRP1 (Figure 6C). Western blot and qRT-PCR analysis confirmed that *miR-148b* caused the decrease in NRP1 expression on the level of both mRNA and protein in PLC/PRF/5 and HuH-7 cells (Figures 6D and 6E).

**Figure 6:**
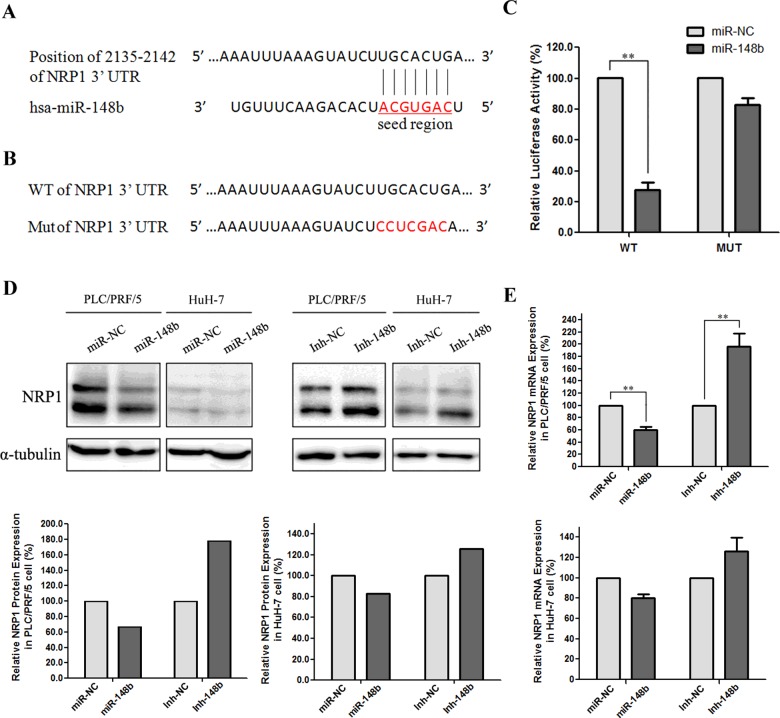
NRP1 was the direct target of *miR-148b* (**A**) The interaction of *miR-148b* and the 3′-UTR of NRP1. (**B**) The mutation of *miR-148b*-binding site of NRP1 3′-UTR. (**C**) Luciferase reporter assays with co-transfection of WT or MUT 3′-UTR of NRP1 and *miR-148b* or miR-NC as indicated. Firefly luciferase activity was normalized by Renilla luciferase activity. (**D**) Western blot analysis of NRP1 protein level in *miR-148b* overexpressed or knocked-down cells of PLC/PRF/5 and HuH-7. E. qRT-PCR analysis of NRP1 mRNA expression in *miR-148b* overexpressed or knocked-down cells of PLC/PRF/5 and HuH-7.

### NRP1 increases expression in SP cells of HCCs

We further investigated the NRP1 expression in SP cells of PLC/PRF/5 and HuH-7 and found that the expression of NRP1 in SP cells was much higher than that in NSP cells of PLC/PRF/5 (9.29% compared with 2.08%) and of HuH-7(1.82% compared with 1.17%; Figure 7A), indicating that NRP1 played a vital role in SP cells of HCC. Moreover, through H&E staining and NRP1 immunohistochemistry, we found that the tumour formed by Inh-148b cells injection showed more tube formation and NRP1 overexpression, as well as excessive cell division and increased nucleo-cytoplasmic ratio, whereas the tumour formed by *miR-148b* cells injection showed the opposite effects (Figure 7B), indicating that *miR-148b* might regulate the tumorigenesis by inhibit the expression of NRP1 and further reduce the formation of SP cells with CSC-like properties.

**Figure 7:**
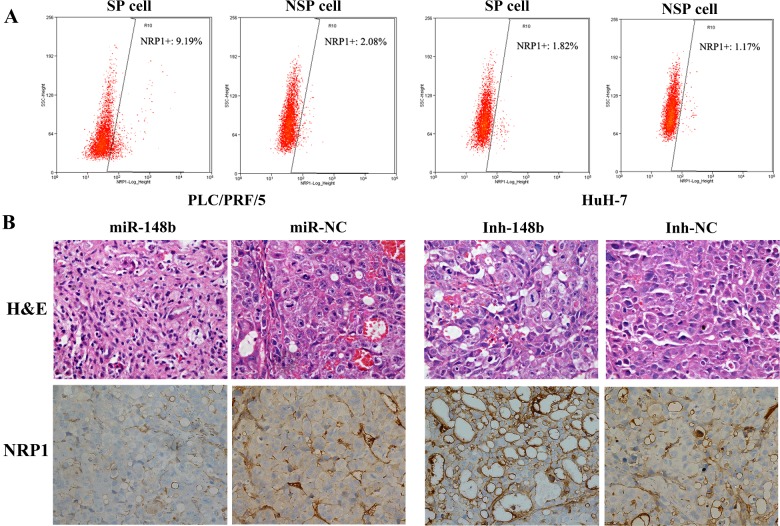
NRP1 was specifically overexpressed in SP cells of PLC/PRF/5 and HuH-7 and under the regulation of *miR-148b* (**A**) NRP1 was specifically overexpressed in SP cells of PLC/PRF/5 and HuH-7. (**B**) H&E staining and anti-NRP1 immunohistochemistry of tumour formed in mice.

## DISCUSSION

In previous study, we have detected *miR-148b* as one of the most down-regulated miRNAs in SP sub-population of PLC/PRF/5 cell line based on the miRNA expression profile screening [15]. In the present study, we further investigated the potential role of *miR-148b* in maintaining SP properties and regulating the progression of tumour initiation and development in HCC. Our data showed that *miR-148b* could inhibit the formation of SP cells, as well as affect cell proliferation, drug resistance, metastasis and angiogenisis by directly targeting to NRP1 in HCC. Additionally, *in vivo* experiment confirmed that *miR-148b* could significantly suppress the tumour growth, suggesting the potential application of *miR-148b* in future clinic therapy of HCC.

Accumulating evidences support that *miR-148b* acts as a tumour-suppressor and down-regulation of *miR-148b* is a frequent event in various tumour types, including liver, gastric, colorectal, pancreatic, breast and lung cancers [17–19,21–24]. Moreover, *miR-148b* is considered as a major co-ordinator of malignancy or an independent prognostic marker since its expression is closely associated with tumour invasion and progression in both breast cancer and liver cancer [19,23,24]. However, the possible role of *miR-148b* in HCSC has not been elucidated. In the present study, we re-verified that *miR-148b* was down-regulated in SP cells of both PLC/PRF/5 and HuH-7 cell lines and further found that the expression level of *miR-148b* could affect the fraction of SP cells significantly as blocking *miR-148b* increased SP percentage, whereas overexpressing *miR-148b* decreased SP percentage both *in vitro* and *in vivo*. These results suggest that *miR-148b* might serve as a potential suppressor in HCC through controlling the SP sub-population, which possess self-renewal and tumour initial ability. Through qRT-PCR, we observed that *miR-148b* inhibition could lead to the up-regulation of CD133, EpCAM, ABCG2 and MMP9 and the down-regulation of TIMP-1, whereas overexpressing *miR-148b* in cells could lead to the almost opposite results. Among these genes, CD133 and EpCAM are proved to be the most important markers for CSC properties in HCC, ABCG2 is especially overexpressed in SP cells as one of the ABC [1]. In addition, MMP9 and TIMP-1 are thought to be involved in tumour metastasis [26]. Our data supported that *miR-148b* might not only play a critical role in maintenance of SP cells with CSC properties, but also associate with tumour metastasis. By coincident, it was reported that the proportion of SP cells was positively related to metastatic potential of parent cells [27]. However, to our surprise, knockdown of *miR-148b* resulted in no remarkable changes in the expression of EMT-related genes, such as BMI 1, SNAI 1, TWIST 1, CDH 1 and CDH 2. Although it was reported that BMI 1 was up-regulated in SP cells in HCC [27], we proposed that *miR-148b* might regulate the maintenance of SP cells in HCC through the distinguished pathway from BMI 1.

Given that CSCs often display higher tolerance to poor environment and chemotherapeutic agents, we tested the influence of *miR-148b* on cell proliferation and drug resistance in the present study and confirmed that *miR-148b* blocking could keep the cell growth rate and increase resistance to cisplatin and 5-FU, the traditional chemotherapeutic drugs used as first-line therapy for HCC. In consonance with our results, Song et al. [16,17] proved that *miR-148b* could inhibit cell proliferation of both gastric cancer and colorectal cancer and Wu et al. [20] revealed that *miR-148b* could enhance the radiosensitivity of non-Hodgkin's lymphoma. Since cell migration and invasion was one of the most important characteristics of CSC, we further investigation confirmed that *miR-148b* inhibition strongly stimulated cell migration and invasion, whereas *miR-148b* overexpression lead cell to reduce the metastasis ability. Meanwhile, we also found that *miR-148b* inhibition enhance the angiogenesis through tube formation assay. In the previous study, *miR-148b* has been found to promote tumour metastasis in pancreatic cancer and breast cancer [19,21]. However, it was the first time to discover the function of *miR-148b* in metastasis and angiogenesis in HCC.

Several genes, such as cholecystokinin B receptor (CCKBR), AMP-activated protein kinase α1 (AMPK α1), carcinoembryonic antigen (CEA) and DNA methyl transferase 3B (DNMT3B), are validated as *miR-148b* target genes and function in regulation of tumour cell growth, tumorigenesis, metastasis and DNA hypomethylation in previous reviews [17,21,22,28]. In our study, NRP1 was confirmed to be a novel direct target of *miR-148b* for the first time. NRP1 is a transmembrane co-receptor for VEGF, TGF-β1, HGF, PDGF-BB and SEMA3 family members. Although it was not essential for receptor signalling, it could enhance response of tyrosine kinase receptors to their ligands [25]. NRP1 was reported to be overexpressed in several human tumour types, including HCC and responsible for tumour initiation and cancer progression, especially for angiogenesis [29,30]. Our data showed that NRP1 is preferentially expressed in SP cells of PLC/PRF/5 and HuH-7, indicating that NRP1 plays a critical role in cancer stem-like cells in HCC. In fact, a link between NRP1 and CSCs has been established in several other cancers, such as breast cancer, skin tumour and glioblastoma multiforme [31–33]. Both in skin tumour and in glioblastoma multiforme, NRP1 was proved to promote the cell self-renewal, stemness and tumorigenicity through VEGF-VEGFR2-NRP1 axis; whereas in breast cancer, nuclear factor of kappa light polypeptide gene enhancer in B cells (NF-kappa B) pathway activation is also involved [31–33]. Although the contribution of NRP1 to regulating the CSC properties of SP cells with needs further investigation, it appears to be a good target for anti-CSC therapy in HCC. Both anti-NRP1 monoclonal antibodies (mAbs) and cell-penetrating peptides of NRP1 have been tested in cancer treatments and shown efficacy in certain cancers [34–36]. Based on our data, raising the *miR-148b* level of patients might be another good therapeutic strategy to treat HCC since the *in vivo* experiment confirmed that *miR-148b* could strongly suppress tumorigenicity, inhibit the tissue NRP1 expression and block the angiogenesis.

In conclusion, our data suggest that *miR-148b* is down-regulated in SP of HCC cell lines PLC/PRF/5 and HuH-7 and affects the CSC properties, including cell proliferation, drug-resistance, metastasis and tumorigenesis. *miR-148b* might act as a CSC suppressor by directly targeting to NRP1 in HCC. Enforced expression of *miR-148b* by nanoparticle conjugates or other methods might supply an effective tool to conquer HCC [37].

## Abbreviations

ABC: ATP-binding cassette proteins
ABCG2: ATP-binding cassette, sub-family G (WHITE), member 2
AMPK α1: AMP-activated protein kinase α1
BMI 1: BMI1 proto-oncogene
CCKBR: cholecystokinin B receptor
CD: cluster of differentiation
CDH: cadherin
CDX2: caudal type homeobox 2
CEA: carcinoembryonic antigen
CMV: cytomegalovirus
CSC: cancer stem cell
DMEM: Dulbecco’s modified Eagle’s medium
DNMT3B: DNA methyltransferase 3B
EMT: epithelial-to-mesenchymal transition
EpCAM: epithelial cell adhesion molecule
GATA6: GATA binding protein 6
HCC: hepatocellular carcinoma
HCSC: hepatic cancer stem cell
H&E: hematoxylin and eosin
HGF: hepatocyte growth factor
mAbs: monoclonal antibodies
MCS: multiple cloning sites
MMP9: matrix metallopeptidase 9
MUT: mutant-type
NF-kappa B: nuclear factor of kappa light polypeptide gene enhancer in B cells
NLK: nemo-like kinase
NRP1: Neuropilin-1
NSP: non-SP
PDCD4: programmed cell death 4
PDGF-BB: the platelet-derived growth factor homodimer
PTEN: phosphatase and tensin homolog
qRT: quantitative real-time
RECK: reversion-inducing-cysteine-rich protein with kazal motifs
RTCA: real-time cellular analysis
SEMA3: Semaphorin-3
SNAI 1: snail family zinc finger 1
SP: side population
TGF-β1: transforming growth factor-β1
TIMP-1: tissue inhibitor of metalloproteinase-1
TWIST 1: twist-related protein 1
VEGF: vascular endothelial growth factor
WNT1: wingless-type MMTV integration site family, member 1
WT: wild-type
